# FEELING AUTHENTIC DURING PLAYING PICKLEBALL IN LATER LIFE: PREDICTING POSITIVE PSYCHOLOGICAL FUNCTIONING

**DOI:** 10.1093/geroni/igz038.1911

**Published:** 2019-11-08

**Authors:** Jungsu Ryu, Jinmoo Heo, Chungsup Lee, Amy Chan Hyung Kim, Kyung Min Kim, Hyunmin Yang

**Affiliations:** 1 Marshall University, Huntington, West Virginia, United States; 2 Yonsei University, Seoul, Korea, Republic of; 3 California State University, Long Beach, Long Beach, California, United States; 4 Florida State University, Tallahassee, Florida, United States; 5 University of Miami, Coral Gables, Florida, United States; 6 Texas A&M University, College Station, Texas, United States

## Abstract

Authenticity, being trustful with oneself, is regarded as a principle predictor of healthy functioning. However, the association between authenticity and psychological functioning has not been examined, even though leisure is an ideal context within which to experience authenticity. Therefore, this study examined the association between authenticity and psychological functioning in older adults playing pickleball. A convenience sample of 112 males and 96 females (64.11±6.56, 50 to 82yrs) was recruited from the 2017 U.S. Open Pickleball Championship which is an annual international pickleball tournament. The participants were asked to fill out a questionnaire primarily asking about their levels of authenticity (4-items) and psychological functioning measured by both perceived stress (4-items) and happiness (single item). The pearson correlation tests found higher levels of authenticity were significantly correlated with being less stressed (r = -.373, p < .01) and happier (r = .203, p < .01). A two-step hierarchical regression was used to determine the unique contribution of authenticity to either perceived stress or happiness, and found that authenticity uniquely contributed to 10% of the variance in perceived stress (F= 4.678, p <.001) and 2.3% of the variance in happiness (F= 3.046, p <.01). These results suggest that feeling authentic in older adults playing pickleball may play an important role in positive psychological functioning.

